# Advancing Community-Based Dementia Support: Insights from South Korea’s Dementia Management System

**DOI:** 10.1093/geroni/igaf122.3698

**Published:** 2025-12-31

**Authors:** Yoon Chung Kim, Takashi Yamashita, Giyeon Kim

**Affiliations:** University of Maryland School of Medicine, Baltimore, Maryland, United States; University of Maryland, Baltimore County, Baltimore, Maryland, United States; Chung-Ang University, Seoul, Republic of Korea

## Abstract

Globally, over 55 million people are living with dementia, with numbers doubling every 20 years. While super-aged societies adopt innovative strategies to support the growing number of people living with dementia (PLWD), gaps remain in systematically sharing practical, scalable models of prevention, diagnosis, and care coordination. This study investigates South Korea’s nationally coordinated, community-based dementia management system. Researchers from the United States and South Korea visited Seoul Metropolitan Center for Dementia (SMCD), participated in a leadership forum with administrative directors, conducted in-depth discussions, and reviewed documents from the National Institute of Dementia (NID) and SMCD. Using a descriptive case study approach, we identified an emphasis on community-based support through five core domains of dementia prevention and awareness, early detection, case registration and management, community resource networking, and online-based support and monitoring. South Korea’s dementia management system demonstrates integration across central (NID), metropolitan/provincial (e.g., SMCD), and local (Dementia Relief Centers; DRC) levels. Notable initiatives include “Ten Million Citizen Memory Friends,” “Memory Café,” and “Dementia-Friendly Villages,” which promote public awareness through community engagement. Local DRCs offer cognitive screening, counseling, diagnostic referrals, and support for PLWD and their families. Dementia service networks are further strengthened through community resources, such as welfare centers, daycare centers, and primary care physicians, via education/training programs and technical assistance. Additionally, mobile applications such as “Wrist Doctor 9988” facilitate health monitoring and prevention of dementia. Our findings highlight valuable insights for developing and promoting dementia-inclusive, community-based models internationally. Future studies are needed to examine dementia management systems across countries.

